# Relationship between body dissatisfaction, insufficient physical activity, and disordered eating behaviors among university students in southern China

**DOI:** 10.1186/s12889-022-14515-9

**Published:** 2022-11-09

**Authors:** Ming Hao, Yifei Fang, Wenjing Yan, Junwang Gu, Yanbin Hao, Chunmei Wu

**Affiliations:** 1 School of Public Health and Health Management, Gannan Medical University, University Park, Rongjiang new area, Ganzhou City, 341000 Jiangxi Province China; 2 Key Laboratory of Prevention and Treatment of Cardiovascular and Cerebrovascular Diseases, Ministry of Education, Gannan Medical University, Ganzhou, 341000 Jiangxi China

**Keywords:** Body dissatisfaction, Exercise habits, Eating behavior, Body image, Southern China

## Abstract

**Background:**

With an increasing incidence of obesity, the relationship between obesity and body image has become a hot research topic worldwide. From high school to university, young people experience changes in their social environment. University students have a high incidence of eating disorders and insufficient physical activity. The purpose of this study was to explore the relationship between body dissatisfaction, insufficient physical activity, and disordered eating behaviors among university students in southern China.

**Methods:**

In total, 1296 university students aged 18–23 years were recruited for this study. The participants completed anthropometric measurements, the Physical Activity Rating scale-3 (PARS-3), and the Chinese-Dutch Eating Behavior Questionnaire (C-DEBQ). The ideal weight and silhouette were reported by university students using a questionnaire.

**Results:**

Compared with men, young women had a higher level of body dissatisfaction. For men, body mass index (BMI; β = 0.76, *P* <  0.01), physical activity score (β = − 0.11, *P* <  0.01), and restrained eating score (β = 0.10, *P* <  0.01) were the significant factors predictive of body dissatisfaction. For women, BMI (β = 0.57, *P* <  0.01), muscle mass (β = 0.12, *P* <  0.01), physical activity score (β = − 0.11, *P* <  0.01), and restrained eating score (β = 0.09, *P* <  0.01) were the significant factors predictive of body dissatisfaction.

**Conclusions:**

University students with high body dissatisfaction had lower physical activity scores and higher restrained eating scores. The data presented here highlight the impact of university students’ body dissatisfaction on physical activity deficiency and disordered eating behaviors in China.

## Background

The increasing rates of overweight and obesity have become issues of international concern. In 2016, far more than 1.9 billion adults older than 18 years were overweight, and over 650 million were obese [1]. With the continuous development of China’s economy, people are paying more and more attention to the health problems caused by obesity [2]. Furthermore, insufficient physical activity deserves attention. Based on a pooled analysis of the results of 358 population-based surveys with 1.9 million respondents, more than a quarter of adults worldwide fail to reach the appropriate level of physical activity [3]. Insufficient physical activity has also been reported in China. Based on the summary results of reports on nutrition and chronic diseases among Chinese (2015), the rate of regular physical exercise among Chinese residents aged 20–69 years was only 18.7% in 2013 [4]. In addition to being an important influencing factor of obesity, insufficient physical activity also increases the risk of cardiovascular and cerebrovascular diseases, anxiety, insomnia, and depression [5–7].

Young individuals try to change their body shape, which leads to eating disorder behaviors. According to a report on the disordered eating behaviors among American high school students, 21.8% of girls and 11.2 of boys had engaged in disordered eating behaviors in the past 30 days [8]. Moreover, a survey of 18-year-old university students in the United States showed a 31% prevalence of disordered eating behaviors [9]. Disordered eating behavior also exists among young Chinese adults. A study conducted in China showed that 2.5% of young Chinese displayed at-risk eating attitudes [10]. Those with high body mass index (BMI) are found to have an increased risk of disordered eating behaviors [10, 11]. Moreover, disordered eating behavior may affect people’s physical health and mental health condition, such as depression [12, 13].

Body image belongs to a psychosocial construct, and was proposed by Schilder in the 1930s as “the picture of our own body which we form in our own mind” [14]. When there is a difference between the actual body and the idealized body, people are dissatisfied with their body shape. Body dissatisfaction is believed to have a negative impact on an individual’s physical and mental health [15]. Body dissatisfaction may lead people to take extreme actions to change their body shape. This includes active vomiting and not eating for extended periods [16, 17]. These extreme behaviors may cause an eating disorder [16, 18]. In addition, high body dissatisfaction is also related to the lack of physical activity habits [19].

University students have a high incidence of eating disorders and insufficient physical activity [20]. From high school to university, young people experience changes in their social environment. They organize their lifestyle independently without parental supervision, and the sudden increase in freedom may make it difficult for them to maintain a healthy lifestyle [21, 22]. Simultaneously, young adults need to adapt to increased independence and academic pressure, making universities a pernicious period for mental health problems [23]. Body dissatisfaction is common among university students [19, 23, 24]. Because the lifestyle of university students has a major effect on the formation of a future healthy lifestyle, it is very important to encourage university students to adopt a healthy lifestyle [25]. However, there are few studies on the relationship between body dissatisfaction, disordered eating behaviors, and physical activity among Chinese university students. The purpose of this study was to explore the relationship between body dissatisfaction, insufficient physical activity, and disordered eating behaviors among Chinese university students.

## Methods

### Participants

A large university was selected in Ganzou city, Jiangxi province, China. This study recruited the study participants through publicity in students’ dormitories and study rooms. As there were very few students younger than 18 years, these students were excluded from the study. This Cross-sectional study surveyed 1296 students (men: 643; women: 653) aged 18–23 years who agreed to participate in this study, conducted from July to December 2021.

### Measures

Height was measured using a portable stadiometer (Seca 213, Germany) with 0.1 cm precision. Body weight (0.1 kg precision), fat percentage, and muscle mass (0.1 kg precision) were measured with a body composition instrument (Tanita BC-610, Japan). BMI (kg/m2) was calculated using height and weight. The body measurements were performed by MH who is experienced in experience. These instruments have been widely used in the world.

### Body image

Regarding body weight dissatisfaction, the ideal weight of young men and women in units (0.1 kg) was recorded using a questionnaire. Subsequently, the ideal BMI was calculated using height and ideal weights, and body dissatisfaction was calculated by combining the actual BMI and ideal BMI values. The difference between the actual BMI and ideal BMI values was the body dissatisfaction score [26].

The participants were asked to complete a set of sex-appropriate silhouettes [27]. The images of the set of silhouettes were numbered between − 7 (fat) and 7 (muscle). Participants were asked to select the ideal silhouette that they most liked to possess and the male and female body figures they considered most attractive.

### Physical activity

The Physical Activity Rating Scale 3 (PARS-3) was used to measure physical activity, a 5-item self-report scale covering duration, intensity, and frequency [28]. Rating of each item on a scale of 1 to 5 and the total score for physical activity (i.e., exercise volume) were computed using the following equation: intensity × (duration − 1) × frequency. The range of the total physical activity score was from 0 to 100. According to the total score, physical activity in this study was categorized into four levels: none, total score ≤ 4; low exercise, total score 5–19; medium exercise, total score 20–42; and high exercise, total score ≥ 43.

### Eating behavior

The Chinese-Dutch Eating Behavior Questionnaire C-DEBQ [29] was used to assess young people’s emotional, external, and restrained overeating style tendencies. Thirteen questions were set for emotional eating, such as “Do you have the desire to eat when you are irritated?”; ten questions were set for external eating, such as “Do you eat more than usual when you see others eating?”; and ten questions were set for restrained eating, such as “Do you find it hard to resist eating delicious foods?”. The 5-point Likert scale is applied to the C-DEBQ, with scores ranging from 1 (never) to 5 (always) from low to high. A higher score indicates a higher tendency for the specific type of overeating.

### Statistical analyses

An independent t-test was performed to examine the differences in the participants’ mean body composition, ideal BMI, body dissatisfaction score, physical activity score, and restrained eating scores by sex. An independent t-test was also used to examine the differences between the silhouette form scores of the most attractive silhouette from the same sex and that from the opposite sex. The Pearson test was used to compare differences between men and women in the BMI and activity level categories. Tukey’s test was performed to examine the differences between the mean values of the three different body types of students’ total scores for physical activity and restrained eating scores according to sex. Body weight dissatisfaction was used as a dependent variable in multiple regression analysis, whereas muscle mass, BMI, total physical activity score, and restrained eating score were included as predictor variables. The variables were chosen according to the stepwise increase-and-decrease method and a threshold *p*-value of 0.20, which was calculated using the likelihood ratio test. All parametric tests were conducted on the premise that the data followed a normal distribution. Statistical significance was set at *P* <  0.05. JMP version 16.0 J (SAS Institute Inc., Cary, NC, USA) was used for all statistical analyses.

## Results

The participants’ characteristics are listed in Table 1. The participants’ ages ranged from 18 to 23 years with a mean (± SD) age of 18.7 ± 1.0 years. Among the students, 19.2% were overweight or obese. The overweight and obesity rates of men were higher than those of women (*P* <  0.05), and 29 percentage of participants were classified as not exercising (Table 1). Nevertheless, the medium and high exercise percentages were 15 and 11%, respectively. The mean values of BMI, ideal BMI, fat percentage, muscle mass, and physical activity in men were higher than those in women (Table 1). The average physical activity score of men was 20.8 (Min: 0; Max: 100), higher than that of women, which was 12.3 (Min: 0; Max: 100) (*P* <  0.05). The scores of each dietary behaviors item were higher for women than for men. The average score of women’s emotional eating was 26.2 (Min:13; Max:65), higher than that of men’s, which was 22.4 (Min: 13; Max: 55) (*P* <  0.05). The average score of women’s external eating was 36.4 (Min: 11; Max: 50), higher than that of men’s, which was 32.0 (Min: 11; Max: 50) (*P* <  0.05). The average score of women’s emotional eating was 28.7 (Min: 11; Max: 50), higher than that of men’s, which was 24.8 (Min: 10; Max: 49) (*P* <  0.05).

**Table 1:** Sample characteristics (*n* = 1296)

	Mean ± SD or n (%)		P
	Male (*n* = 643)	Female (*n* = 653)	
BMI (kg/m^2^)	22.1 ± 3.7	21.2 ± 3.1	< 0.01
Fat%	16.1 ± 6.7	27.7 ± 6.0	< 0.01
Muscle mass (g)	50.4 ± 7.6	36.0 ± 4.0	< 0.01
Ideal BMI (kg/m^2^)	21.2 ± 2.2	19.3 ± 2.4	< 0.01
Body dissatisfaction(kg/m^2^)	0.8 ± 3.5	2.0 ± 2.9	< 0.01
BMI category			
Underweight	95 (15)	106 (16)	< 0.01
Normal	390 (60)	458 (70)	
Overweight & Obesity	158 (25)	89 (14)	
Physical activity score	20.8 ± 20.4	12.3 ± 16.1	< 0.01
Activity level category			
No exercise	138 (21)	242 (37)	< 0.01
Low exercise	256 (40)	307 (47)	
Medium exercise	141 (22)	59 (9)	
High exercise	108 (16)	45 (7)	
Eating behavior			
Emotional eating score	22.4 ± 9.4	26.2 ± 9.8	< 0.01
External eating score	32.0 ± 8.0	36.4 ± 7.1	< 0.01
Restrained eating score	24.8 ± 8.0	28.7 ± 7.3	< 0.01

*BMI* body mass index. The significance of differences between male and female students was determined by t-test (for quantitative variables) or by Pearson analyses (qualitative variables)

**Fig. 1:**
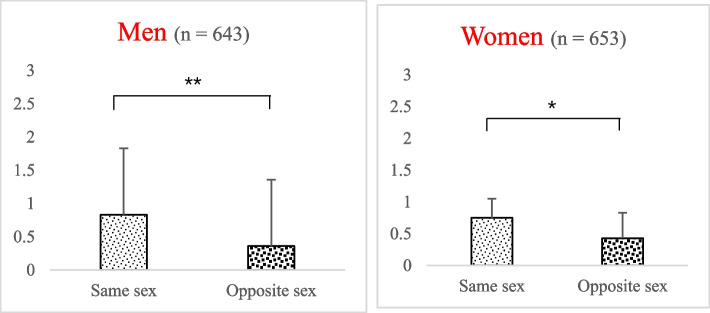
Ideal silhouette of the same sex and the opposite sex students. ^*^ t-test, *P* < 0.05; ^**^ t-test, *P* < 0.01

From the male perspective, the most attractive figure of women was thinner than the most attractive figure of men, as shown in Fig. 1. From the female perspective, the most attractive figure of men was thinner than the most attractive figure of women, as shown in Fig. 1.

**Fig. 2:**
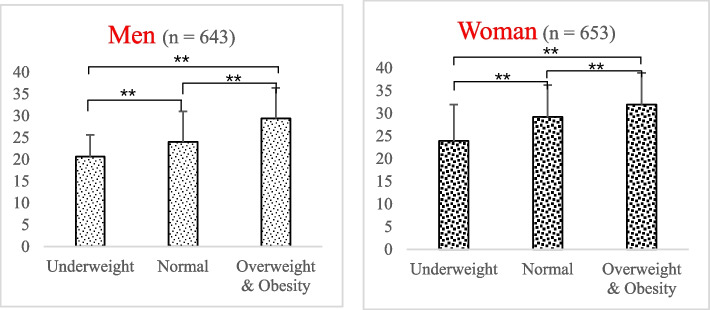
Restrained eating score of university students with different BMI category. ^**^ Tukey, *P* < 0.01

The restrained eating scores of the students in different BMI categories are shown in Fig. 2. For both men and women, the restrained eating score increased with an increase in the BMI.

Table 2 shown the results for multiple regression analysis of the factors contributing to body dissatisfaction for each sex. For men, BMI (β = 0.76, *P* <  0.01), physical activity score (β = − 0.11, *P* <  0.01), and restrained eating score (β = 0.10, *P* <  0.01) were the significant factors predictive of body dissatisfaction. For women, BMI (β = 0.57, *P* <  0.01), muscle mass (β = 0.12, *P* <  0.01), physical activity score (β = − 0.11, *P* <  0.01), and restrained eating score (β = 0.09, *P* <  0.01) were the significant factors predictive of body dissatisfaction.

**Table 2:** Factors that contributed to body dissatisfaction among university students

	β	*t*	VIF	*P*
Men ^‡^				
BMI (kg/m2)	0.76	31.28	1.22	< 0.01
Physical activity score	−0.11	−5.12	1.04	< 0.01
Restrained eating score	0.10	4.21	1.24	< 0.01
Emotional eating score	−0.04	−1.97	1.06	NS
Women^#^				
BMI (kg/m2)	0.57	16.80	1.47	< 0.01
Muscle mass (g)	0.12	3.72	1.41	< 0.01
Physical activity score	−0.11	−3.76	1.02	< 0.01
Restrained eating score	0.09	2.82	1.01	< 0.01
Emotional eating score	0.05	1.82	1.02	NS

*BMI* body mass index, *VIF* variance inflation factor

^‡^R^2^: 0.69; *P* < 0.01; Root Mean Square Error (RMSE): 1.93

^#^R^2^: 0.49; *P* < 0.01; RMSE: 2.06

## Discussion

The results of this study showed that the mean BMI was higher for men than for women; however, the level of body dissatisfaction was higher among women than among men (Table 1). These findings replicate previous research findings [30, 31]. With the constant advertising in the media in the Western countries and even in China, the belief that a woman’s thinness is beautiful is deeply rooted in the public’s minds [32]. In today’s modern society, a slim and graceful figure is considered one of the conditions for women to get more benefits [33]. According to a pioneering study that examined the body image of men and women, women have a higher requirement for a slim body than men; thus, more women try to lose weight than men [34, 35]. Furthermore, behaviors in relation to body monitoring and body change among women in the Chinese society are more pronounced [36]. In this study, a higher level of dissatisfaction with women’s bodies was presumed to reflect the socio-cultural background described above.

Studies have shown that men and women have different reasons for body dissatisfaction [32, 37, 38]. Whereas men want a strong body [38], women recognize that their slim figure is considered beautiful [32]. However, the current study found that the higher the body dissatisfaction level of young women, the higher the ideal silhouette scores (Table 2). This shows that young women are satisfied with having a slim body and with the pursuit of muscle acquisition [39]. A study of 388 women aged 17–35 years in Australia also found a change in women’s ideal bodies [40]. However, a muscular body requires exercise for a long period, which is more difficult to achieve than that required for a slim body [39]. Therefore, the pursuit of muscle is not unique to men and an important factor affecting women’s body dissatisfaction.

Research shows that in the desire for a slim figure, peer perceptions play an important role in body dissatisfaction [41]. With respect to the desire for muscle among both men and women, the ideal silhouette for young people of the opposite sex was thinner than that for young people of the same sex (Fig. 1). The desire for muscles may be related to self-perception. Interestingly, we found that body dissatisfaction among young men was also related to their ideal silhouette of the opposite sex. Well-educated women in Asia are increasingly pursuing education and career opportunities instead of marriage and motherhood [42]. Moreover, the male to female sex ratio in China has risen from 1 to 1.18 [43]. The declining dominance of men in marriage and relationships may be an important reason that men’s ideal heterosexual silhouettes are slimmer and their body dissatisfaction is higher.

Moderate-intensity and high-intensity physical activity is recommended by the World Health Organization to reduce the potential risk of chronic disease [6]. The results of this study showed that 29 percentage of young people lacked physical activity habits and only 43% of young people had low physical activity. A lack of physical activity habits among young people in southern China is common. A survey of 650 university students in Guangzhou also found that 28% of young people lacked physical activity habits and 45% of young people were reported to often engage in low physical activity [28]. Exercise intervention is the most direct way to improve low exercise levels in young people. However, simple exercise interventions lack long-term effects on the increase in the exercise level [44].

This study proposes psychological motivations that may aid in improving young people’s physical activity levels (Table 2). The results showed that both men and women had higher levels of body dissatisfaction and lower physical activity scores (Table 2). Currently, it is debatable whether body dissatisfaction results in resistance to exercise or motivates participation in physical activity. For example, a survey of 1044 university students from 17 countries on body dissatisfaction and physical activity relationships supports that body dissatisfaction is an important motivation for young people to participate in sports [45]. However, compared to participating in physical activity for health, participating in physical activity to improve appearance is not sustainable [19]. Concomitantly, body dissatisfaction leads to the avoidance of physical activity [19]. For example, people with a higher level of body dissatisfaction are more likely to think that physical activity is embarrassing and they will avoid sports and activities involving motor skills to avoid being considered unattractive [46]. Therefore, reducing body dissatisfaction and cultivating health awareness are of great significance for improving the current situation of low physical activity among young people.

The higher the body dissatisfaction level among university students, the higher their restrained eating score (Table 2). A study of more than 18,500 university students from 22 countries showed that both men and women in Asian countries were more likely than those in other regions to try to lose weight, indicating that Asian university students had a high level of body dissatisfaction [47]. Body dissatisfaction is considered one of the important reasons for eating disorders [48]. Like other Asian countries, Chinese university students also have body dissatisfaction [49] and may lead to dieting (Table 2). The situation of obese university students may be more prominent. To have a slim body, overweight women are more likely to perform restricted fasting or skip meals and engage in dietary restriction behaviors than their normal-weight peers [50]. A retrospective study of 179 young people aged 12–22 years in the United States found that adolescents with a history of overweight or obesity accounted for a large proportion of people who controlled their weight through dietary restrictions [51]. The results of this study support those of previous studies. Young people with obesity scored higher on restricted eating. Obese young people are more likely to have eating disorders because others do not believe that they are really dieting, and they may even be praised for unhealthy eating behaviors [52]. With the continuous increase in obesity in China and worldwide, the problem of dietary restriction may become more serious in the future. To prevent obesity, sustainable healthy eating and physical activity patterns rather than dieting should be encouraged.

The results of this study emphasized the relationship between body dissatisfaction, BMI, and food restrictions in young people. The higher the level of body dissatisfaction, the higher the restrained eating score, which may aggravate obesity. Restricted eating may negatively affect obesity. First, the effect of restricted eating on body improvement is usually short-term [44]. Second, the compensatory relationship between restrictive behavior and overeating leads to obesity and other diseases [21]. Therefore, establishing a positive body image will help prevent and improve disordered eating in young people. Studies have proven that eating disorders can be improved effectively by body dissatisfaction interventions. Moreover, the effect of improving body dissatisfaction is better than that of direct intervention in dietary behavior [12]. The findings of this study, combined with the results of other previous studies [52], clarify the association between body dissatisfaction and eating disorders and emphasize the importance of improving body dissatisfaction in young people.

### Limitations

This study had some limitations. First, the small sample size in this study included only young people aged 18–23 years. Moreover, as this was a cross-sectional study, causal inferences should not be made. In addition, only southern China was surveyed in this study. Therefore, future work should address body image in northern China for comparison.

## Conclusions

Our findings indicated that young women had a higher level of body dissatisfaction than men. The data presented here highlight the impact of university students’ body dissatisfaction in China on physical activity deficiency and disordered eating behaviors.

## Data Availability

The datasets used and analyzed during the current study are available from the corresponding author on reasonable request.

## Abbreviations

BMI: Body mass index
C-DEBQ: Chinese-Dutch Eating Behavior Questionnaire
PARS-3: Physical Activity Rating Scale 3
